# *Areca catechu* L. Extract Inhibits Colorectal Cancer Tumor Growth by Modulating Cell Apoptosis and Autophagy

**DOI:** 10.3390/cimb47020128

**Published:** 2025-02-17

**Authors:** Meng-Hsiu Tsai, Chang-Han Chen, Chien-Lin Chen, Mei-Hsien Lee, Li-Ching Wu, Yi-Chiung Hsu, Chao-Yang Hsiao, Chang-Ti Lee, Kuo-Li Pi, Li-Jen Su

**Affiliations:** 1 Department of Biomedical Science and Engineering, National Central University, Taoyuan 320317, Taiwan; billy1990914@gmail.com (M.-H.T.);; 2 Department of Applied Chemistry, Graduate Institute of Biomedicine and Biomedical Technology, National Chi Nan University, Nantou County 345301, Taiwan; 3 Department of Medical Research, Taichung Veterans General Hospital, Taichung 407219, Taiwan; 4 IHMED Reproductive Center, Taipei 106028, Taiwan; 5 Graduated Institute of Pharmacognosy, Taipei Medical University, Taipei 110301, Taiwan; 6 Division of Rheumatology, Allergy and Immunology, Department of Internal Medicine, Chang Gung Memorial Hospital, Taoyuan 333423, Taiwan; 7 Department of Chinese Medicine, Taipei Tzu Chi Hospital, Buddhist Tzu Chi Medical Foundation, New Taipei 231016, Taiwan; 8 Graduate Institute of History, National Central University, Taoyuan 320317, Taiwan; 9 Education and Research Center for Technology Assisted Substance Abuse Prevention and Management, National Central University, Taoyuan 320317, Taiwan; 10 Core Facilities for High Throughput Experimental Analysis, Department of Biomedical Science and Engineering, National Central University, Taoyuan 320317, Taiwan

**Keywords:** apoptosis, *Areca catechu* L., autophagy, colorectal cancer, MAPK pathway, PI3K/AKT pathway

## Abstract

Colorectal cancer (CRC) is a common cancer globally, and chemotherapy often causes severe complications, necessitating effective drugs with minimal side effects. As *Areca catechu* L. extract (ACE) is a Traditional Chinese Medicine that contains numerous active compounds with anticancer effects, in this study, the Cell Counting Kit-8 (CCK-8) assay was used to determine ACE’s effect on CRC cell lines, revealing that it significantly inhibits CoLo320DM and HCT116 cells. In vivo experiments with NU-Foxn1nu mice indicated that ACE inhibits tumor growth, while a flow cytometry assay revealed that higher ACE concentrations increased cell apoptosis and ROS levels. Next-generation sequencing (NGS) showed that ACE increases the fold changes in apoptosis, DNA damage, and autophagy-related genes while inhibiting the fold changes in cell proliferation and Wnt signaling pathway genes. We conducted Western blotting to confirm these findings. Overall, ACE demonstrates potential as a drug candidate by promoting apoptosis and autophagy, and significantly reducing cell viability and tumor growth, thus offering a new approach for effective colorectal cancer treatment with minimal side effects.

## 1. Introduction

Colorectal cancer (CRC) is one of the most prevalent cancers worldwide [1], with its incidence rising in recent years. It accounts for approximately 10% of all cancer cases and is the second-leading cause of cancer-related mortality [2,3]. The risk of developing CRC is influenced by several factors, including family history, an aging population, dietary habits in developed countries, obesity, lack of moderate exercise, smoking, and alcohol consumption [4]. CRC screening has proven to be effective in significantly reducing both the incidence and mortality rates of this disease. Currently, various invasive and non-invasive screening methods are available for the detection and early diagnosis of colorectal cancer. These include fecal occult blood tests, multi-target stool DNA tests, fecal immunochemical tests, serum SEPT9 circulating tumor DNA tests, colonoscopy, computed tomography colonography, and sigmoidoscopy [5,6]. The management of CRC typically involves a multimodal approach that includes surgical intervention along with adjuvant therapies such as chemotherapy, radiation therapy, and targeted therapy [7,8,9].

In this context, Traditional Chinese Medicine (TCM), which has been used for centuries in East Asia, has gained increasing attention as a potential adjunctive therapy in cancer treatment. TCM has been shown to alleviate cancer symptoms, reduce the side effects of chemotherapy, and improve patients’ quality of life [10]. Additionally, certain TCM formulations have demonstrated the ability to overcome drug resistance in cancer cells, thereby enhancing the effectiveness of conventional chemotherapy [11,12]. For example, *Astragalus membranaceus* (Fisch.) Bunge, *Panax ginseng* C.A.Mey., and *Scutellaria baicalensis* Georgi have also been reported to inhibit tumor growth, improve sensitivity to chemotherapy and radiotherapy, modulate immune system function, and reduce treatment-related side effects [13].

Among the various TCMs investigated for their therapeutic potential, *Areca catechu* L. (AC), commonly known as betel nut, has gained particular attention for its effects on colorectal health. Its bioactive components have traditionally been used as laxatives and anthelmintics, primarily for the treatment of gastrointestinal diseases caused by parasitic infections [14]. AC extract (ACE) contains a diverse range of bioactive compounds, with approximately 59 different identified chemical constituents, including alkaloids, flavonoids, tannins, triterpenes, steroids, and fatty acids, all of which contribute to its biological effects. Several isolated compounds or constituents of ACE have exhibited pharmacological activities, including antiparasitic, antidepressant, antifatigue, antioxidant, antibacterial, antifungal, antihypertensive, anti-inflammatory, analgesic, antiallergic, and digestive-promoting effects, as well as the ability to inhibit platelet aggregation and regulate blood glucose and lipid levels [15,16,17]. There is evidence suggesting that ACE can suppress hepatocellular carcinoma (HCC) progression by inducing autophagy and apoptosis via reactive oxygen species (ROS) generation [18].

We utilized next-generation sequencing (NGS) and a bioinformatics analysis to better understand the molecular mechanisms underlying the therapeutic effects of ACE. NGS has revolutionized genomic research, enabling the comprehensive analysis of genetic alterations, drug resistance mechanisms, and cancer subtypes [19]. It has also become an invaluable tool in personalized medicine, offering insights into the complex pathways involved in cancer progression and helping to optimize treatment strategies [20,21]. In oncology, NGS facilitates for the analysis of drug resistance mechanisms and the classification of cancer subtypes [21]. Bioinformatics tools such as the Database for Annotation, Visualization and Integrated Discovery (DAVID) can be employed to analyze and interpret large-scale data, identifying key signaling pathways and gene expression changes associated with ACE treatment [22,23].

This study aimed to investigate the anticancer properties of ACE in CRC, with a particular focus on its ability to induce apoptosis and modulate oxidative stress and cell survival pathways. To achieve this, we employed CCK-8 assays to assess cell viability, flow cytometry to evaluate Annexin V expression and ROS levels, and Western blotting to analyze key apoptotic and autophagic markers. Additionally, an NGS analysis was performed to explore potential molecular mechanisms and signaling pathways associated with ACE treatment. By elucidating the molecular basis of ACE-induced apoptosis and oxidative stress, this study provides insights into its potential as a therapeutic candidate for CRC.

## 2. Materials and Methods

### 2.1. Cell Culture

HCT116 and CoLo320DM cell lines were procured from the Bioresource Collection and Research Center (BCRC, Hsinchu, Taiwan). HCT116 and CoLo320DM were cultured in McCoy’s 5A medium and RPMI 1640 medium (Gibco (Thermo Fisher Scientific), Carlsbad, CA, USA), respectively, supplemented with 10% fetal bovine serum (FBS) (Hyclone, Logan, UT, USA) and 1% penicillin–streptomycin solution (P/S) (Hyclone, Logan, UT, USA). The cells were maintained at 37 °C in a humidified atmosphere containing 5% CO_2_.

### 2.2. Extract of AC

The AC was obtained from Young Jin Chinese Herbal Medicine Store (Taichung City, Taiwan; Batch No.: 202112). To prepare the crude extract, 200 g of dried AC particles was immersed in 3000 mL of deionized water overnight, followed by decoction at 95 °C for six hours. The resulting extract was filtered through filter paper and concentrated under reduced pressure using a rotary evaporator. The concentrated extract was then lyophilized into a powder and stored at −20 °C until further use.

### 2.3. Treatment with Crude Extracts of Traditional Chinese Medicines

CoLo320DM was treated with the crude extract of Traditional Chinese Medicine at a concentration of 250 μg/mL for 72 h, while HCT116 was treated with the crude extract at a concentration of 400 μg/mL for 72 h.

### 2.4. Cell Viability Assay

Cell viability was assessed using the Cell Counting Kit-8 (CCK-8) assay. Briefly, CoLo320DM and HCT116 cells were seeded in 96-well plates at a density of 5 × 10^3^ cells per well and allowed to adhere for 24 h. Following the initial 24 h incubation, the cells were treated with crude extracts of Traditional Chinese Medicine at concentrations of 250 and 400 μg/mL, respectively, for 72 h. After treatment, the medium was replaced with 50 μL of fresh medium containing 1/10 volume of CCK-8 solution, followed by incubation at 37 °C for 2 h. Absorbance was measured at 450 nm to determine cell viability via a Mobi UV/Vis Microplate Spectrophotometer.

### 2.5. Xenograft Experiment

This study utilized male NU-Foxn1nu mice (BioLASCO Taiwan Co., Ltd., Taipei, Taiwan) as an animal model for subcutaneous xenograft experiments. Approximately 1 × 10^7^ CoLo320DM cells were subcutaneously injected into the right flank of each mouse in a volume of 100 μL PBS. The mice were randomly assigned to two groups: group 1 (*n* = 5) received 100 μL RO H_2_O via gavage, while group 2 (*n* = 5) received 100 μL of ACE via gavage. ACE was administered at a dose of 300 mg/kg/day, with oral administration beginning 7 days after subcutaneous injection. After 28 days of oral administration, the mice were euthanized using CO_2_. This study was conducted in accordance with the Declaration of Helsinki and approved by the Institutional Animal Care and Use Committee (approval date: 7 May 2021 and approval number: Ncu-110-011).

### 2.6. Annexin V Analysis

Cell apoptosis assays were performed according to the manufacturer’s instructions and analyzed using the Muse^®^ Cell Analyzer (Merck Millipore, Rahway, NJ, USA). CoLo320DM and HCT116 cells were treated with varying concentrations of ACE for 72 h, after which they were collected in 50 μL of phosphate-buffered saline (PBS) and incubated with 50 μL of Muse™ Annexin V and Dead Cell Reagent and 1% fetal bovine serum (FBS). The cells were then subjected to detection using the Muse instrument after 20 min of incubation at room temperature.

### 2.7. Oxidative Stress Kit Analysis

Cellular oxidative stress assays were conducted in accordance with the manufacturer’s instructions using the LUMINEX Muse^®^ Oxidative Stress Kit (Merck Millipore, Rahway, NJ, USA). The Muse Oxidative Stress Reagent was first diluted to an intermediate solution at a ratio of 1:100 with 1× Assay Buffer, and further diluted to a working solution at a ratio of 1:80 with 1× Assay Buffer. CoLo320DM cells were treated with varying concentrations of ACE for 72 h and then prepared in 1× Assay Buffer at a concentration of 1 × 10^6^ to 1 × 10^7^ cells/mL for incubation with the working solution. Subsequently, 10 μL of the cell suspension was added to 190 μL of the working solution and incubated at 37 °C for 30 min prior to detection using the Muse instrument.

### 2.8. Autophagy (Autophagosome) Detection Assay

Cells were seeded in a 96-well plate at a density of 10,000 cells/well. Following the user manual, after the cells adhered to the bottom, the old medium was removed, and a 0.1 mM DAPRed reagent was diluted to 0.1 µM with a serum-free medium and added to the wells for 30 min. Subsequently, different concentrations of ACE were added and incubated for 12 h, with or without a 2 h pretreatment with NAC (10 mM). Finally, images were captured using a fluorescence microscope at 200× magnification, with an exposure time of 60 ms.

### 2.9. NGS Data Analysis

After sequencing, the indexed FASTQ files were subjected to a quality control (QC) assessment using FASTQC. The QC-passed FASTQ files were then aligned to the reference genome using HISAT2. The resulting aligned BAM files were processed with FeatureCounts to quantify the number of reads per gene. Subsequently, DESeq2 was employed to calculate the transcripts per million (TPM) values for each gene under different experimental conditions. After obtaining the TPM values for all genes, we first filtered out genes with TPM < 1. The specific process is as follows: For upregulated genes, we strictly selected those whose TPM values increased with the ACE dose and had a TPM value greater than 1 in the 250 µg/mL group. Conversely, for downregulated genes, we strictly selected those whose TPM values decreased with the ACE dose and had a TPM value greater than 1 in the control group. The genes were further classified as upregulated (with a fold change > 2 between the 250 µg/mL and control groups or a fold change = ∞) or downregulated (with a fold change < 0.5 between the 250 µg/mL and control groups or a fold change = 0). The gene symbols were then analyzed to generate gene ontology and KEGG pathway data via DAVID.

### 2.10. Gene Ontology Image Generation

Following the input of upregulated and downregulated genes into the DAVID platform, we obtained gene ontology (GO) biological process results for both sets of genes. The data were subsequently downloaded and organized using Excel. We retained the GO terms, gene count, gene ratio, and *p*-value for the biological processes, while excluding non-significant entries (*p*-value > 0.05). The remaining data were then sorted in descending order based on gene count. The top 20 entries were selected to create an enrichment plot using the ggplot2 package in R version 4.3.1. [24].

### 2.11. Protein Extraction

After CoLo320DM cells were treated with varying concentrations of ACE for 72 h, the cells were collected and lysed using a specialized lysis buffer for 30 min. The resulting supernatant was obtained through centrifugation at 13,000× *g* rpm for 30 min. Protein quantification was performed using the Pierce™ BCA Protein Assay Kit (Thermo Fisher Scientific, Waltham, MA, USA).

### 2.12. Western Blotting

The proteins were separated via sodium dodecyl sulfate–polyacrylamide gel electrophoresis (SDS-PAGE) and subsequently transferred to a polyvinylidene fluoride (PVDF) membrane. The membrane was then blocked using 5% blocking milk, followed by incubation with primary antibodies against Phosphatidylinositol-4,5-Bisphosphate 3-Kinase Catalytic Subunit Alpha (PIK3CA, 1:1000), Phosphoinositide-3-Kinase Regulatory Subunit 1 (PIK3R1, 1:1000), p-AKT Serine/Threonine Kinase 1 (p-AKT1, 1:1000), BCL2 Apoptosis Regulator (BCL2, 1:1000), Autophagy-Related 5 (ATG5, 1:1000), Microtubule Associated Protein 1 Light Chain 3 Alpha/Beta (LC3I/II, 1:1000), Autophagy-Related 3 (ATG3, 1:1000), Autophagy-Related 16 Like 1 (ATG16L1, 1:1000), and Glyceraldehyde-3-Phosphate Dehydrogenase (GAPDH, 1:40,000) for specific protein detection. The primary antibodies against PIK3CA, PIK3R1, p-AKT1, BCL2, ATG5, LC3I/II, ATG3, and ATG16L1 were purchased from Cell Signaling Technology, Danvers, MA, USA, while the primary antibody against GAPDH was purchased from Jackson ImmunoResearch Laboratories, Inc., West Grove, PA, USA. For Western blot quantification, the protein expression level at each concentration was first normalized to the GAPDH level of the corresponding well. The normalized value was then divided by the normalized value of the control group to obtain the final ratio.

### 2.13. Statistical Analysis

A statistical analysis was performed to compare the results of three independent experiments using a one-tailed Student’s *t*-test. A significance level of *p* < 0.05 was set to denote statistical significance between the two compared groups.

## 3. Results

### 3.1. ACE-Induced CRC Cell Line Apoptosis

**Figure 1:**
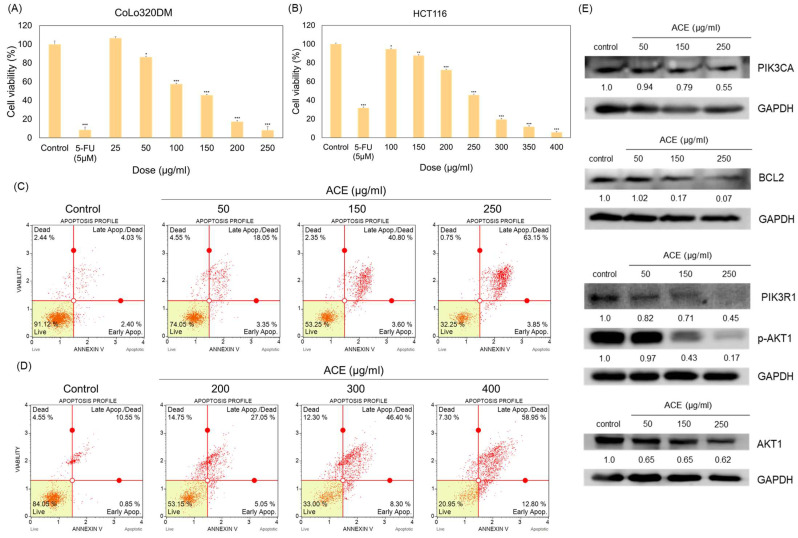
Colorectal cancer cell lines treated with ACE. (**A**) CoLo320DM and (**B**) HCT116 treated with ACE at different doses for 72 h, with 5-FU (5 μM) as the positive control. Apoptosis levels in (**C**) CoLo320DM and (**D**) HCT116 were measured after treatment with ACE at different doses for 72 h using the Annexin V assay. (**E**) The expression levels of apoptosis-related proteins, including PIK3CA, PIK3R1, AKT1, p-AKT1, and BCL2, were measured in CoLo320DM cells treated in the control condition or with ACE at concentrations of 50, 150, and 250 μg/mL for 72 h, where the protein expression level at each concentration was first normalized to the GAPDH level of the corresponding well. The normalized value was then divided by the normalized value of the control group to obtain the final ratio. The presented data represent the mean ± standard deviation (SD) of three independent experiments conducted in triplicate, with statistical significance denoted as * *p* < 0.05, ** *p* < 0.01, and *** *p* < 0.001.

CRC cell lines CoLo320DM (Figure S1A) and HCT116 (Figure S1C) were exposed to TCM extracts for 72 h at concentrations of 250 μg/mL and 400 μg/mL to evaluate their pro-apoptosis potential. Our CCK-8 assay results demonstrated that ACE exhibited a significantly stronger inhibition of cell viability compared to other TCM extracts tested, including *Saposhnikovia divaricata* (Turcz.) Schischk., *Houttuynia cordata* Thunb., *Angelica dahurica* (Hoffm.) Benth. & Hook.f. ex Franch. & Sav., *Saussurea costus* (Falc.) *Lipsch.*, *Fritillaria thunbergii* Miq., *Benincasa hispida* (Thunb.) Cogn., *Tussilago farfara* L., *Schisandra chinensis* (Turcz.) Baill., *Asparagus cochinchinensis* (Lour.) Merr., *Zingiber officinale* Roscoe, *Eriobotrya japonica* (Thunb.) Lindl., *Aster taiwanensis* Kitam., and *Eupatorium japonicum* Thunb. Moreover, ACE demonstrated a marked inhibition of cell viability across all three CRC cell lines. Subsequent testing revealed that the IC_50_ values for CoLo320DM (Figure 1A) and HCT116 (Figure 1B) were approximately 135.4 μg/mL and 235.1 μg/mL, respectively. Annexin V levels were measured to confirm ACE-induced apoptosis. In CoLo320DM (Figure 1C) and HCT116 (Figure 1D) cells, treatment with varying doses of ACE led to a dose-dependent increase in the percentage of late apoptotic cells compared to the control. The percentage distributions of viable (Annexin V−/7-Aminoactinomycin D (7-AAD)−), early apoptotic (Annexin V+/7-AAD−), and late apoptotic (Annexin V+/7-AAD+) cells were quantified and labeled within each respective quadrant in the flow cytometry plots. The detailed cell percentages at different apoptosis stages for CoLo320DM and HCT116 are presented in Table 1. Furthermore, we conducted experiments using LoVo cells, and the results were similar to those observed in CoLo320DM and HCT116 cell lines (Figure S1B,D,E). These findings indicate that ACE induces apoptosis in all three CRC cell lines. However, subsequent experiments primarily focused on CoLo320DM cells, as they are a poorly differentiated CRC cell line with higher malignancy, aggressive growth characteristics, and an association with a poor prognosis [25,26]. These features make CoLo320DM a clinically relevant model for studying the cytotoxic and apoptotic effects of ACE, particularly in highly aggressive CRC subtypes. Our initial findings demonstrated that ACE effectively induces apoptosis in CRC cell lines. To further validate these results, CoLo320DM cells were treated in the control condition or with 50 μg/mL, 150 μg/mL, or 250 μg/mL of ACE for 72 h, after which the expression levels of apoptosis-related proteins were analyzed via Western blotting. Western blot results indicated that the expression levels of PIK3CA, PIK3R1, AKT1, p-AKT1, and BCL-2 decreased progressively with increasing concentrations of ACE, demonstrating a dose-dependent suppression. The protein band intensities were normalized to GAPDH within each lane, and the relative expression levels were determined by comparing each ACE-treated group to the control group. The quantified ratios are presented beneath each corresponding lane (Figure 1E).

### 3.2. ACE Treatment Inhibited CRC Tumor Growth in Nude Mice

To evaluate the in vivo anticancer effects of ACE, we employed a nude mouse model to assess its impact on CRC tumor progression. The results showed that the tumor size in the ACE-treated group was not significantly smaller than that in the control group (Figure S2A). Additionally, a slight reduction in tumor growth rate was observed in the ACE-treated group compared to the control group, but only between days 21 and 28 (Figure S2B), with detailed tumor volumes presented in Figure S2C. No significant changes in body weight were observed in ACE-treated mice. Furthermore, a histological evaluation of the liver (Figure S2D, left) and kidney (Figure S2D, right) using hematoxylin and eosin (H&E) staining revealed no signs of inflammatory cell infiltration, further confirming its safety profile. Additionally, a TUNEL analysis and immunohistochemistry (IHC) were performed to examine tumor tissues. The TUNEL staining showed no detectable evidence of DNA fragmentation within the tumors. The IHC staining with an LGR-5 antibody revealed a decrease in LGR-5 expression in ACE-treated tumors (Figure S3), which, although in line with our expectations, was not statistically significant. Overall, the in vivo effects of ACE were less pronounced than those observed in vitro, demonstrating only a slight inhibitory effect on tumor growth.

### 3.3. Biological Processes Induced or Inhibited by ACE in CoLo320DM Cells

**Figure 2:**
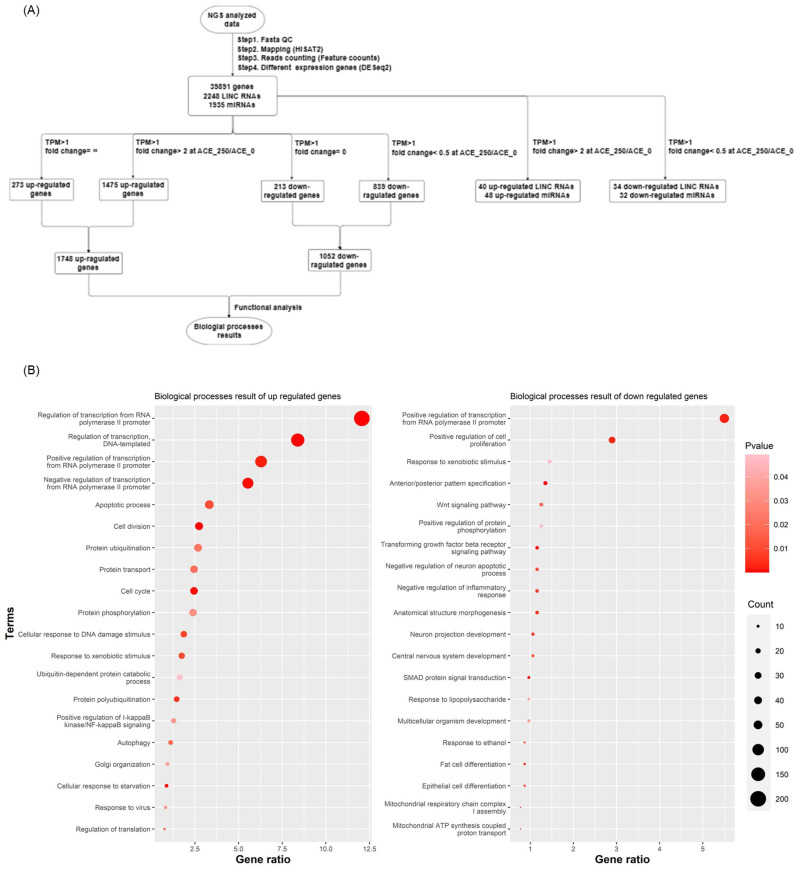
A gene ontology analysis was conducted on the upregulated and downregulated genes identified from the NGS data of CoLo320DM cells treated in the control condition or with ACE at various concentrations (50, 150, and 250 μg/mL) for 72 h. (**A**) A flow chart illustrating the steps involved in the analysis of NGS data. A biological process enrichment analysis was performed separately for (**B**) the upregulated genes (left) and the downregulated genes (right).

After 72 h of treatment in the control condition and with varying concentrations of ACE (50, 150, and 250 μg/mL), mRNA from CoLo320DM cells was subjected to NGS. The NGS analysis workflow involved quality control using FASTA QC, alignment with HISAT2, quantification with FeatureCounts, and a differential expression analysis using DESeq2. This comprehensive analysis yielded a total of 39,891 genes, 2247 long non-coding RNAs (lncRNAs), and 1936 microRNAs (miRNAs). These transcripts were further categorized into upregulated genes (1748) and downregulated genes (1052) based on fold change criteria (fold change ≥ 2 or fold change = ∞ for upregulated genes, and fold change ≤ 0.5 or fold change = 0 for downregulated genes), followed by a gene ontology (GO) analysis (Figure 2A). The GO analysis identified biological processes such as apoptosis, protein ubiquitination, and the cellular response to DNA damage stimuli among the upregulated genes (Figure 2B, left), while the downregulated genes were enriched in processes related to cell cycle regulation, translation, Wnt signaling, and cell proliferation (Figure 2B, right).

**Figure 3:**
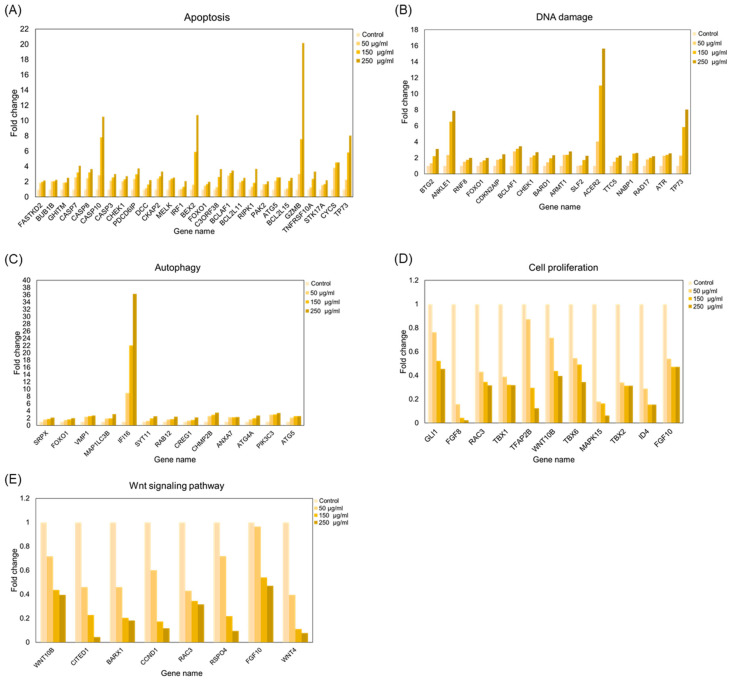
mRNA expression of target genes in candidate signaling pathways. The upregulated gene expression pathways are associated with (**A**) apoptosis, (**B**) DNA damage, and (**C**) autophagy, while the downregulated gene expression pathways are related to (**D**) cell proliferation and (**E**) Wnt signaling.

In addition to the GO analysis, we further examined the expression levels of key genes involved in critical cellular processes. The results showed that genes related to cell apoptosis (Figure 3A), the DNA damage response (Figure 3B), and autophagy (Figure 3C) exhibited an increased expression level with higher ACE concentrations compared to the control group. Conversely, genes involved in cell proliferation (Figure 3D) and the Wnt signaling pathway (Figure 3E) showed a concentration-dependent decrease in expression. These findings not only corroborate our earlier observations but also provide deeper insights into the molecular mechanisms by which ACE regulates gene expression at the mRNA level.

The results were further supported by a gene ontology analysis using the Database for Annotation, Visualization and Integrated Discovery (DAVID). Upregulated and downregulated gene sets were analyzed separately, and non-significant terms (*p*-value > 0.05) were removed. The remaining terms were ranked by gene count, and detailed data are provided in Supplementary Data S4 (Excel file). Overall, these analyses highlight the impact of ACE on processes such as apoptosis, DNA damage response, and autophagy, as well as the suppression of pathways related to cell proliferation and Wnt signaling.

### 3.4. ACE Induced Cell Apoptosis and ROS Generation in CoLo320DM Cells

**Figure 4:**
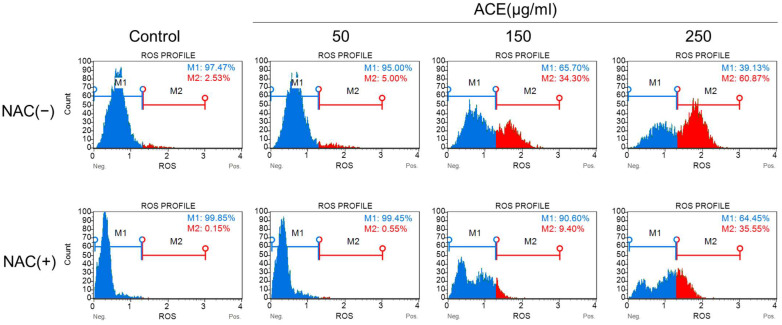
The ROS level of CoLo320Dm treated with ACE at different concentrations for 72 h with or without NAC (10 mM) pretreated for 2 h. After 72 h of treatment in the control condition or with 50, 150 or 250 μg/mL of ACE, the intracellular ROS levels in CoLo320DM cells increased in a concentration-dependent manner. Furthermore, in the group pretreated with NAC for 2 h, intracellular ROS levels were reduced compared to the group without NAC pretreatment; however, the ROS levels still exhibited a concentration-dependent increase with ACE.

**Figure 5:**
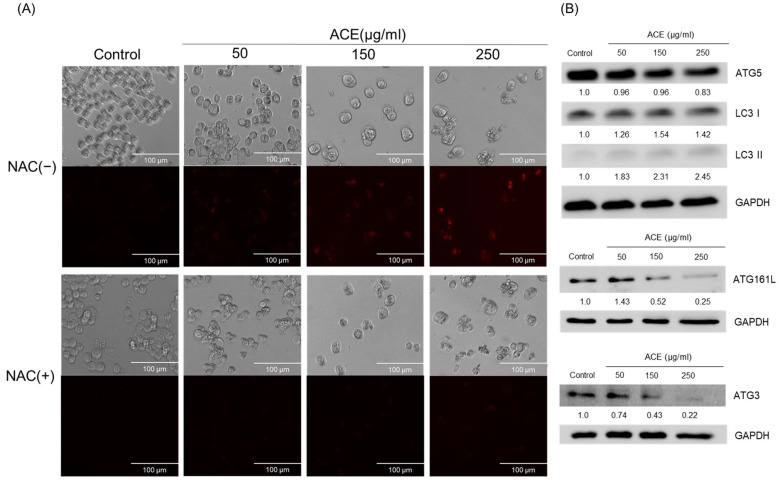
Assessment of the autophagic response in CoLo320DM cells following treatment with varying concentrations of ACE. (**A**) The autophagy image of CoLo320DM treated in the control condition or with ACE at concentrations of 50, 150, and 250 μg/mL for 12 h with or without NAC. (**B**) The expression levels of autophagy-related proteins, including ATG3, ATG5, ATG161L, and LC3I/II, were measured in CoLo320DM cells treated in the control condition or with ACE at concentrations of 50, 150, and 250 μg/mL for 72 h, where the protein expression level at each concentration was first normalized to the GAPDH level of the corresponding well. The normalized value was then divided by the normalized value of the control group to obtain the final ratio.

We initially measured the intracellular ROS levels to further elucidate the mechanism of ACE-induced apoptosis. The results demonstrated a concentration-dependent increase in ROS levels in CoLo320DM cells treated with 150 and 250 μg/mL of ACE compared to the control. In parallel, an additional experiment was conducted where cells were pretreated with N-acetylcysteine (NAC) for 2 h prior to treatment with varying concentrations of ACE. The findings revealed that NAC pretreatment effectively inhibited the generation of intracellular ROS (Figure 4). The excessive accumulation of intracellular ROS could cause severe damage to proteins, nucleic acids, lipids, membranes, and organelles, ultimately leading to the initiation of cell death cascades, particularly apoptosis [27]. Given that ACE treatment induces ROS generation in CoLo320DM cells, we further employed DAPRed staining to evaluate intracellular autophagic activity. DAPRed is a fluorescence-based autophagy detection dye that specifically labels autophagosomes and autolysosomes, emitting red fluorescence in an acidic environment. Our results demonstrated that a 12 h treatment with varying concentrations of ACE triggered autophagic activity in the absence of NAC pretreatment, with stronger red fluorescence observed at higher ACE concentrations. In contrast, NAC-pretreated cells exhibited no significant autophagic response following 12 h ACE treatment, and red fluorescence signals were not detected (Figure 5A). CoLo320DM cells were treated in the control condition or with 50 μg/mL, 150 μg/mL, and 250 μg/mL of ACE for 12 h, with or without NAC pretreatment. Compared to the control group, the expression of autophagy-related protein LC3 I/II increased with higher concentrations of ACE. In contrast, the expression of ATG5 showed a marginal decrease at 50, 150, and 250 μg/mL compared to the control. Notably, the expression level of ATG3 decreased with increasing concentrations of ACE. The expression level of ATG16 L1 increased after treatment with low concentrations of ACE (50 μg/mL) but decreased after treatment with high concentrations of the extract (150 and 250 μg/mL). The protein band intensities were normalized to GAPDH within each lane, and the relative expression levels were determined by comparing each ACE-treated group with the control group. The quantified ratios are presented beneath each corresponding lane (Figure 5B). We also examined tumor tissues using IHC. IHC staining with an LC3-II antibody showed a reduction in LC3-II expression in tumors treated with ACE (Figure S5). While the results are consistent with our expectations, the reduction was not pronounced and did not reach statistical significance. Autophagy is generally considered a critical cell survival process, activated under conditions of nutrient deficiency and cellular stress to maintain cellular integrity and homeostasis. However, increasing evidence highlights the dual role of autophagy, as it is also associated with the promotion of cell death. The specific impact of autophagy on cell death depends on the cell phenotype and contextual cues [28,29].

### 3.5. LncRNAs and miRNAs Induced or Inhibited by ACE in CoLo320DM Cells

Over the past decade, studies have highlighted the ability of long non-coding RNAs (lncRNAs) and microRNAs (miRNAs) to regulate mRNA expression, thereby exerting broad cellular effects, including the modulation of oncogenic and tumor-suppressive signaling pathways [30]. Based on these findings, our research employed NGS to analyze the lncRNA and miRNA expression profiles in CoLo320DM cells following ACE treatment.

We identified nine upregulated miRNAs and three upregulated lncRNAs, which were considered tumor suppressor genes [31,32,33,34,35,36,37,38,39,40,41,42,43,44,45,46,47,48,49]. Conversely, we also identified four downregulated miRNAs and eight downregulated lncRNAs, which were associated with oncogenic functions [50,51,52,53,54,55,56,57,58,59,60,61,62,63,64,65,66,67,68,69,70,71,72,73,74,75,76,77,78,79]. Among the upregulated molecules, miR-4746 [32], miR-491 [33], miR-646 [39,40], miR-8075 [45], and LINC01752 [49] were reported to play key roles in CRC progression. Similarly, among the downregulated molecules, LINC00467 [62], LINC00538 [55], and LINC00963 [78] were implicated in CRC development. Additionally, Table 2 presents a comprehensive list of upregulated tumor suppressor genes and downregulated oncogenes, further demonstrating the anticancer potential of ACE.

## 4. Discussion

There are various treatment options and combinations currently available for CRC in clinical practice; however, significant challenges remain. While targeted therapies have demonstrated efficacy, their high costs render them inaccessible to many patients. Additionally, chemotherapy remains a standard treatment, but its non-specific cytotoxic effects on both malignant and normal cells often lead to severe side effects [80,81]. Given these challenges, there is an urgent need to explore safe and viable alternative therapeutic strategies. According to previous studies, PHY906, a formula derived from four herbal components—*Scutellaria baicalensis* Georgi, *Glycyrrhiza uralensis* Fisch., *Paeonia lactiflora* Pall., and *Ziziphus jujuba* Mill.— has been used as an adjuvant therapy for irinotecan-based chemotherapy in CRC [82,83]. In this context, ACE, another TCM, emerges as a promising candidate for CRC treatment, warranting further investigation into its therapeutic potential. In this study, ACE exhibited significant cytotoxic effects on CRC cells in vitro, supporting its potential as an anticancer agent. To further evaluate its effects, we selected CoLo320DM, a poorly differentiated CRC cell line with high malignancy and a poor prognosis, for subsequent experiments [25,26]. Notably, although ACE inhibited tumor growth in vivo, it did not exhibit a significant direct cytotoxic effect on tumors. This outcome is speculated to be a consequence of oral administration, which may have reduced ACE’s bioavailability and cytotoxic efficacy. Future animal studies should therefore consider intraperitoneal injection as an alternative route of administration to enhance ACE’s therapeutic effect. From a safety perspective, no hepatotoxicity or nephrotoxicity was observed, and no significant changes in body weight or food intake were detected in the mice during the experiment. Compared to conventional chemotherapy drugs, ACE appears to have a relatively lower impact on quality of life, further supporting its potential as a therapeutic agent for CRC.

The Annexin V assay results revealed a concentration-dependent increase in apoptosis in CoLo320DM cells following ACE treatment. The Western blot analysis demonstrated that ACE treatment led to reduced expression levels of anti-apoptotic proteins, such as PIK3CA, PIK3R1, p-AKT1, and BCL2. Furthermore, the NGS analysis revealed increased expression levels of pro-apoptotic genes after ACE treatment, including CASP3, CASP7, CASP8, BID, and CYCS. These findings collectively suggest that ACE induces apoptosis through both the mitochondrial [84,85,86] and PI3K/AKT pathways [87,88,89].

A subsequent analysis using an ROS detection kit revealed a dose-dependent increase in intracellular ROS levels in CoLo320DM cells following ACE treatment. Conversely, pretreatment with the antioxidant NAC resulted in a marked reduction in intracellular ROS levels. Notably, the elevated ROS levels triggered an enhancement in autophagic flux, as demonstrated through fluorescence staining with the DAPRed reagent, where fluorescence intensity was positively correlated with the ACE dosage. This observation was further corroborated via the upregulation of the expression of the essential autophagy-related protein LC3 I/II [90,91,92]. Although autophagy typically acts as a pro-survival mechanism by inhibiting apoptosis, the excessive or prolonged activation of autophagy can lead to autophagic cell death [28].

**Figure 6:**
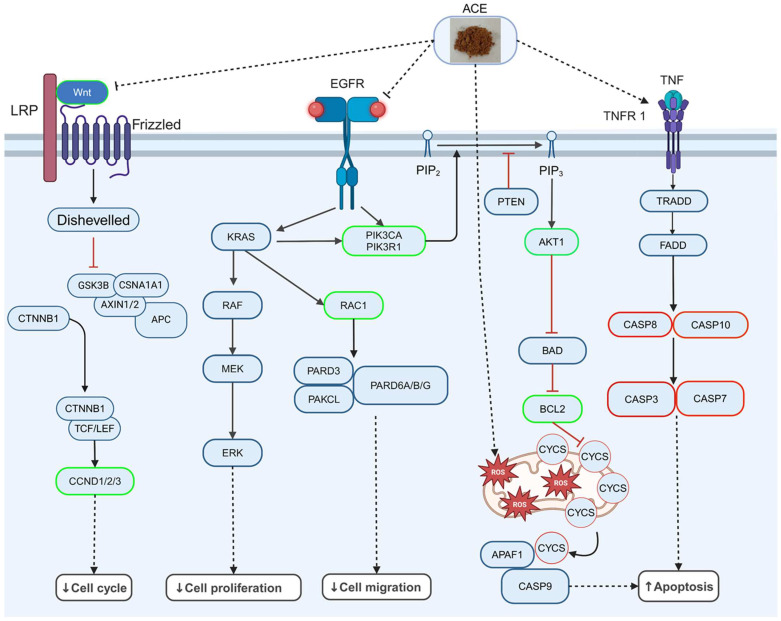
Signaling pathways induced and inhibited by ACE. Based on the results of the KEGG signaling pathway, we found that ACE inhibits survival, the cell cycle, cell proliferation, and cell migration by suppressing the Wnt, PI3K/AKT, and MAPK signaling pathways. At the same time, it induces ROS generation and promotes apoptosis through the caspase signaling pathway. The red border indicates a TMP fold change greater than 2, while the green border indicates a TMP fold change of less than 0.5.

When analyzing biological processes and signaling pathways, it can be observed that ACE not only promotes cell apoptosis but also inhibits the cell cycle, cell survival, cell proliferation, and cell migration, all of which are factors that contribute to tumor progression [93,94]. The signaling pathways involved in these processes include the Wnt, PI3K/AKT, MAPK, RAS/RAF, and RAS/RAC signaling pathways [95,96,97,98,99,100,101,102,103]. These pathways have commonly been associated with cancer progression in previous studies, and many anticancer drugs have been developed to target them [39,104,105,106,107,108,109,110,111,112,113]. The PI3K/AKT and MAPK signaling pathways can also induce angiogenesis [114,115]. However, no specific signaling pathway inhibiting angiogenesis was identified after ACE treatment. The genes associated with these biological processes were integrated using the KEGG pathway, culminating in the generation of a comprehensive signaling pathway diagram (Figure 6) [116]. This illustration reveals that the cellular mechanisms involved not only promote apoptosis but also inhibit cell survival, proliferation, cell cycle progression, and migration. The integrated signaling pathway diagram primarily reflects differential gene expression, with only the bottom section depicting proteins related to autophagy and apoptosis that correspond to gene expression. It is anticipated that subsequent research on the deconstruction of ACE components will identify distinct constituents with inhibitory effects on various oncogenic pathways.

Extensive research in the literature has demonstrated that dysregulation in various cellular signaling pathways contributes to the malignant phenotype observed in CRC [117]. In this study, we utilized two CRC cell lines, namely HCT116, which represents primary carcinoma (Duke’s type A) with an epithelial morphology that adheres to the culture dish, and CoLo320DM, a metastatic CRC cell line (Duke’s type C) characterized by a rounded morphology. The significant differences in gene expression between primary and metastatic carcinomas contribute to the challenges associated with treating metastatic cancer, which is often more difficult to cure [118,119]. Our findings indicate that ACE exhibits cytotoxic effects against both CoLo320DM and HCT116 in vitro. Given that ACE contains a diverse array of active components, some of these may specifically target either primary or metastatic carcinomas. Future studies will focus on isolating and characterizing these active components, with the aim of developing novel therapeutic agents for the effective treatment of CRC.

The therapeutic application of AC has been highly controversial, primarily due to its potential carcinogenicity regarding oral cancer. There is a well-established association between oral cancer and betel nut chewing. However, our study demonstrates that ACE, when used under controlled conditions, exhibits significant anticancer properties in CRC without inducing toxicity. In the context of TCM, AC is subjected to drying and water extraction to obtain ACE, which is subsequently utilized as a purgative or for the treatment of gastrointestinal parasitic infections [19]. The recent literature has increasingly highlighted the pharmacologically active constituents present in ACE, including flavonoids, tannins, steroids, and fatty acids. These bioactive compounds exhibit a range of therapeutic properties such as cardiovascular and cerebrovascular protection, anti-inflammatory effects, anti-obesity effects, and organ-protective effects [120,121,122,123,124]. Through our rigorous experimental validation, we have demonstrated that ACE induces both apoptosis and autophagy in CoLo320DM cells. Moving forward, we aim to further fractionate ACE to identify small-molecule compounds with potential therapeutic efficacy against CRC.

## 5. Conclusions

In this study, we elucidated the anticancer properties of ACE and confirmed its ability to induce apoptosis. Through an NGS data analysis, we tested the hypothesis that ACE induces DNA damage by increasing intracellular oxidative stress, leading to autophagy and apoptosis via the caspase signaling pathway. Subsequent experiments demonstrated that ACE significantly elevated oxidative stress levels in CoLo320DM cells while also inducing autophagic activity. Furthermore, we found that ACE inhibits cell survival and promotes apoptosis by suppressing the PI3K/AKT signaling pathway. In in vivo experiments, ACE exhibited a slight inhibitory effect on tumor growth without causing significant changes in body weight or food intake in mice. Additionally, a histological examination of H&E-stained liver and kidney tissue sections revealed no evidence of ACE-induced damage. These findings suggest that ACE is a promising anticancer agent with significant therapeutic potential for further development.

## Figures and Tables

**Table 1:** The cell percentages at different apoptosis stages.

Cell Line	Dose (μg/mL)	Live (%)	Apoptosis (%)		
			Early	Late	Total
CoLo320DM	Control	91.1	2.4	4.0	6.4
	50	74.1	3.4	18.0	21.4
	150	53.3	3.6	40.1	43.7
	250	32.3	3.9	63.2	67.1
HCT116	Control	84.1	0.9	10.6	11.5
	200	53.2	5.1	27.1	32.2
	300	33.0	8.3	46.4	54.7
	400	21.0	12.8	59.0	71.8

**Table 2:** LncRNA and miRNA candidate list for ACE treatment targets in CoLo320DM.

Type	Regulatory	Symbol	Cancer ^▲^	Property
miRNA	Up	MIR4482	PCa [31]	Tumor suppressor gene
		MIR4746	CRC [32]	Tumor suppressor gene
		MIR491	BC, BCa, CC, CRC, ESCC, GBM, GC, HCC, HNSC, LC, LKM, OSC, OVCA, PC, PCa, RBM, TC, TGC [33]	Tumor suppressor gene
		MIR548AN	PC [34]	Tumor suppressor gene
		MIR635	GC [35], NSCLC [36]	Tumor suppressor gene
		MIR643	GC [37], OSCC [38]	Tumor suppressor gene
		MIR646	CRC [39,40], NSCLC [42], PTC [41]	Tumor suppressor gene
		MIR7152	HCC [43], HNSCC [44]	Tumor suppressor gene
		MIR8075	CC [46], CRC [45]	Tumor suppressor gene
	Down	MIR4530	PC [50]	Oncogene
		MIR23B	LC [51], PCa [52]	Oncogene
		MIR200CHG	BC [53]	Oncogene
		MIRLET7BHG	HCC [54]	Oncogene
LncRNA	Up	LINC00921	TNBC [47]	Tumor suppressor gene
		LINC01504	NSCLC [48]	Tumor suppressor gene
		LINC01752	CRC [49]	Tumor suppressor gene
	Down	LINC00304	PCa [59]	Oncogene
		LINC00467	BC [60], BCa [61], CRC [62], GC [63], GM [64], HCC [65], HNSC [66], LUAD [67], OS [68], PCa [69]	Oncogene
		LINC00538	CRC [55]	Oncogene
		LINC00852	GC [70], HCC [71], NSCLC [72], OC [73], OSC [74], PCa [75]	Oncogene
		LINC00896	COAD [76], LUSC [77]	Oncogene
		LINC00963	BC, CRC, ESCC, GC, HCC, NSCLC, OC OSCC [78]	Oncogene
		LINC01711	ESCC [56], GBM [57], RCC [58]	Oncogene
		LINC02298	KIRP, LUAD [79]	Oncogene

▲: BC, breast cancer; CC, cervical cancer; COAD, colon adenocarcinoma; CRC, colorectal cancer; ESCC, esophageal squamous cell carcinoma; GBM, glioblastoma; GC, gastric cancer; GM, glioma; HCC, hepatocellular carcinoma; HNSC, head and neck squamous cell carcinoma; KIRP, kidney renal papillary cell carcinoma; LC, lung cancer; LKM, leukemia; LUAD, lung adenocarcinoma; NSCLC, non-small-cell lung cancer; LSCC, laryngeal squamous cell carcinoma; OC, ovarian cancer; OSC, osteosarcoma; OSCC, oral squamous cell carcinoma; PTC, papillary thyroid cancer; PC, pancreatic cancer; PCa, prostate cancer; RBM, retinoblastoma; TC, thyroid cancer; TGC, tongue cancer; TNBC, triple-negative breast cancer.

## Data Availability

The original contributions presented in this study are included in the article/Supplementary Material. Further inquiries can be directed to the corresponding author.

## Supplementary Materials

The following supporting information can be downloaded at https://www.mdpi.com/article/10.3390/cimb47020128/s1.
