# Supplementary material for: Areca catechu L. Extract Inhibits Colorectal Cancer Tumor Growth by Modulating Cell Apoptosis and Autophagy
Source: Curr Issues Mol Biol. 2025 Feb 17;47(2):128. doi: 10.3390/cimb47020128 (PMC11854706; doi:10.3390/cimb47020128)
Supplement: Supplementary file 1 [file cimb-47-00128-s001.zip › supplementary file/Figure S1.pdf]

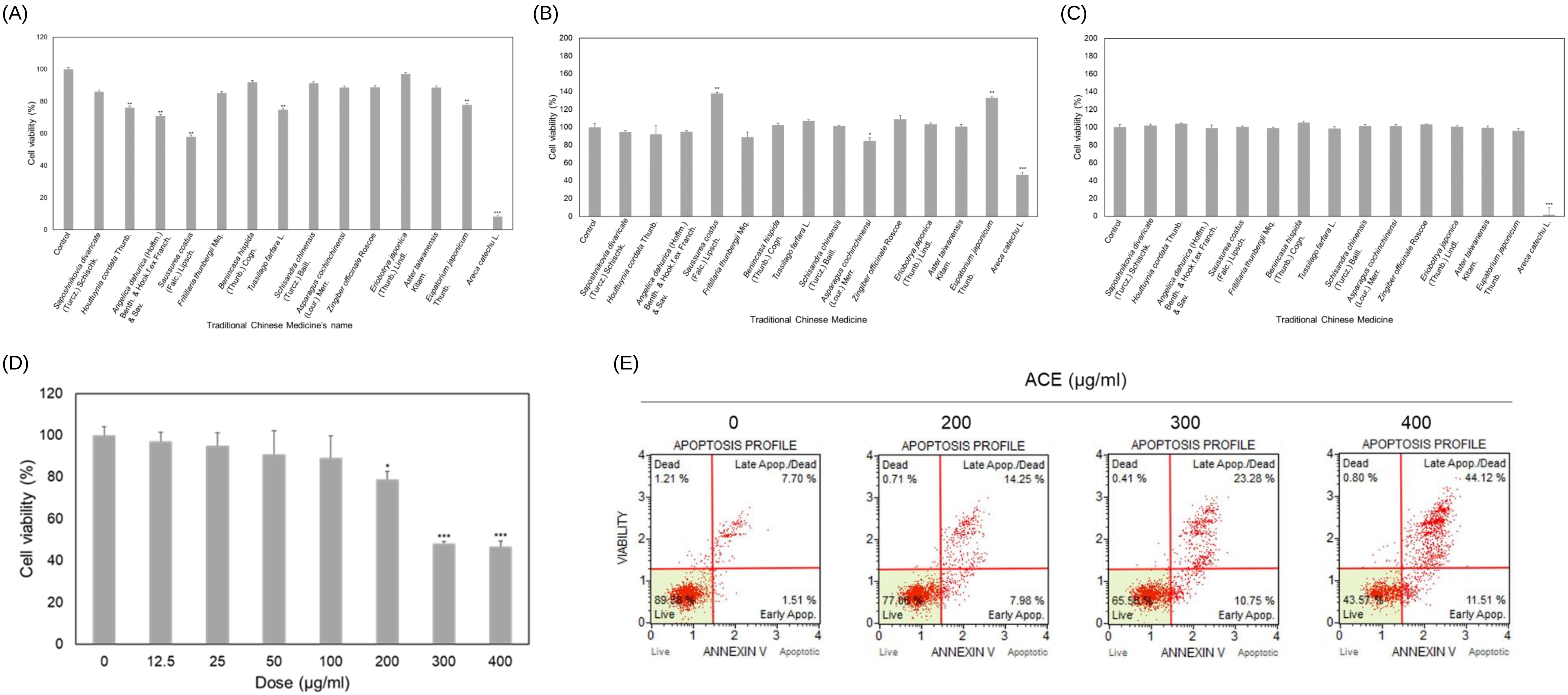

Figure S1. Colorectal cancer cell lines treat with TCMs. (A)CoLo320DM, (B) LoVo and (C) HCT116 treat with TCMs for 72 hours at 250 μg/ml 400 μg/ml, respectively. (D) LoVo treated with ACE at different doses for 72 hours. (E) Apoptosis levels in LoVo was measured after treatment with ACE at different doses for 72 hours using the Annexin V assay. The presented data represents the mean±standard deviation (SD) of three independent experiments conducted in triplicates, with statistical significance denoted as * p< 0.05, ** p< 0.01, and *** p< 0.001.
