# Supplementary material for: Areca catechu L. Extract Inhibits Colorectal Cancer Tumor Growth by Modulating Cell Apoptosis and Autophagy
Source: Curr Issues Mol Biol. 2025 Feb 17;47(2):128. doi: 10.3390/cimb47020128 (PMC11854706; doi:10.3390/cimb47020128)
Supplement: Supplementary file 1 [file cimb-47-00128-s001.zip › supplementary file/Figure S2.pdf]

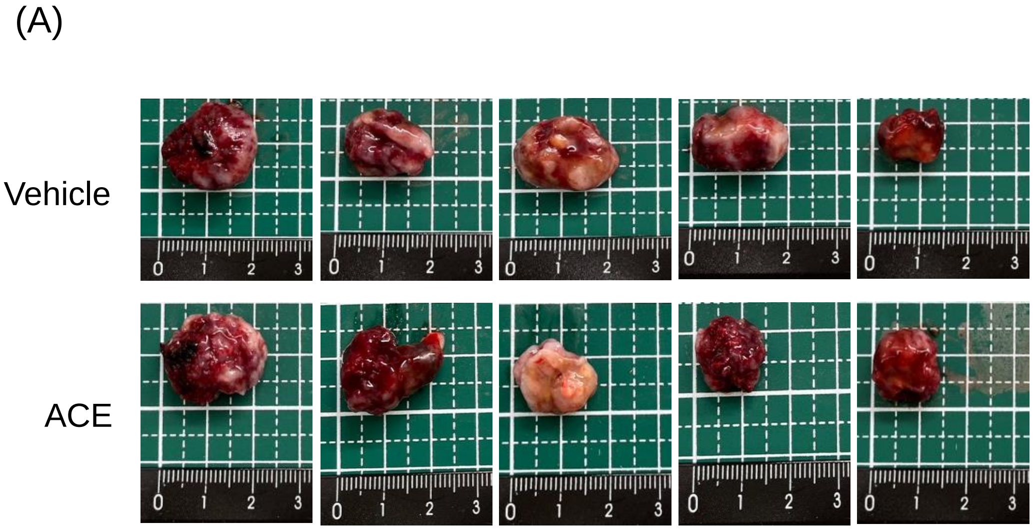

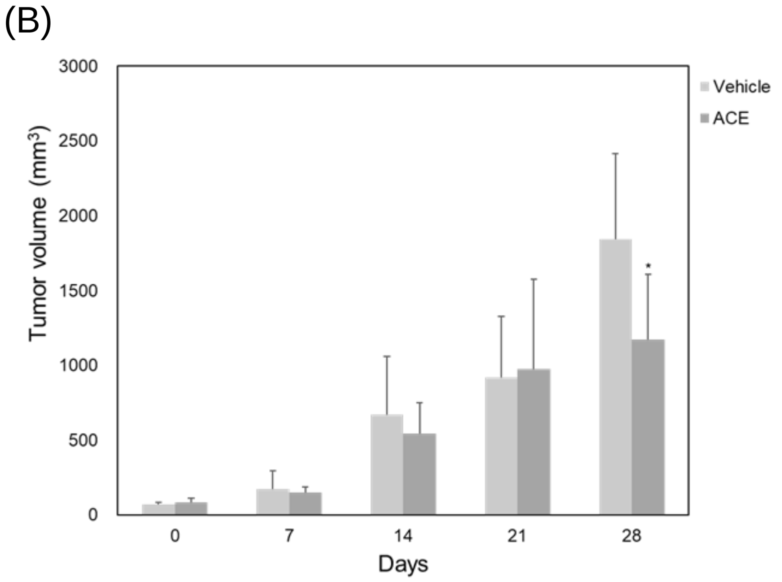

(C)

Tumor volume (mm³)	Time (day)				
	0	7	14	21	28
Vehicle	70.0±6.2	172.0±54.6	670.0±174.1	918.0±184.2	1841.5±255.8
ACE	85.0±13.0	152.0±16.3	543.0±93.0	975.0±268.3	1172.0±195.7 *

*: ACE group compared with Vehicle at Day 28, p-value< 0.05

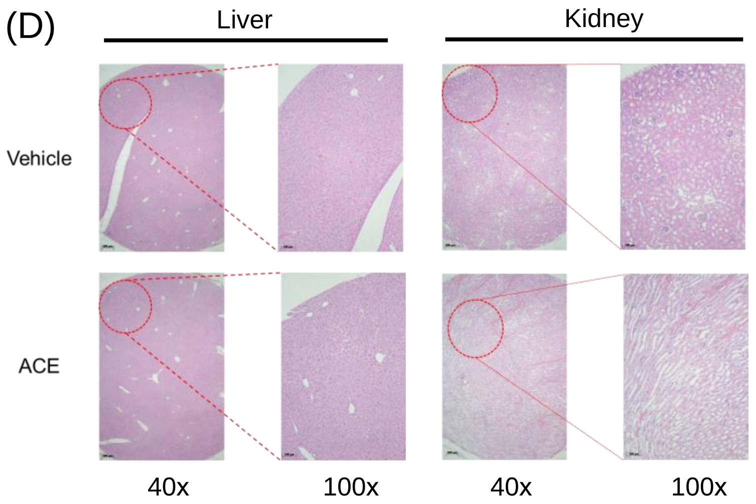

Figure S2. ACE exhibited inhibitory effects on tumor growth in CoLo320DM xenograft nude mice. (A) Representative images comparing tumors treated with vehicle and ACE. (B) Tumor volume measurements of vehicle- and ACE-treated tumors over time. The pre-sented data represent the SE of five independent experiments conducted in quintuplicate, with statistical significance indicated as * P< 0.05. (C) Average tumor volumes of vehicle-treated and ACE-treated groups (mean±standard error). (D) H&E staining of liver (left) and kidney (right) tissues observed at 40x and 100x magnifications.
