# Supplementary material for: Areca catechu L. Extract Inhibits Colorectal Cancer Tumor Growth by Modulating Cell Apoptosis and Autophagy
Source: Curr Issues Mol Biol. 2025 Feb 17;47(2):128. doi: 10.3390/cimb47020128 (PMC11854706; doi:10.3390/cimb47020128)
Supplement: Supplementary file 1 [file cimb-47-00128-s001.zip › supplementary file/Figure S3.pdf]

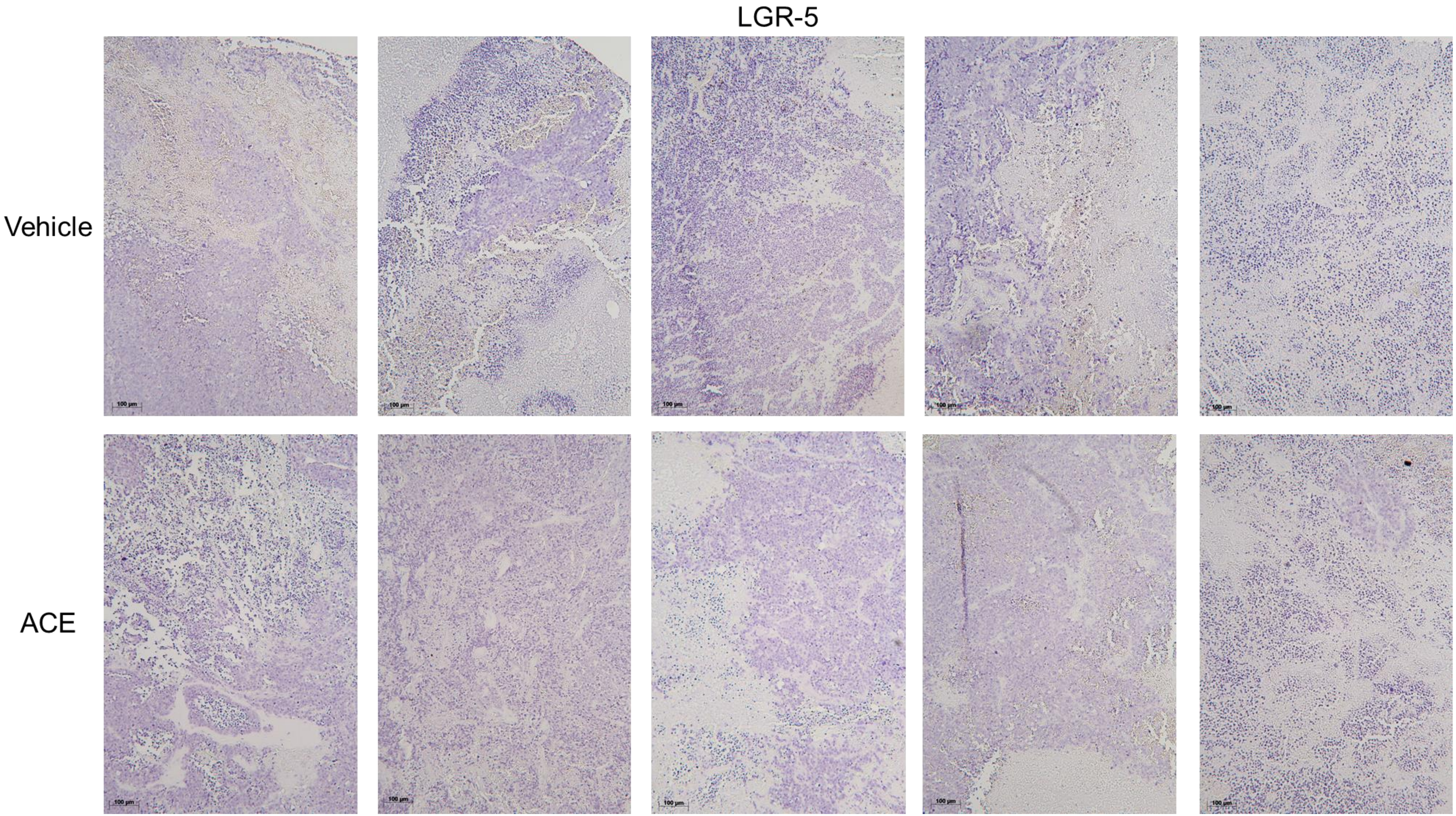

Figure S3. IHC staining using an LGR-5 antibody. The result showed a reduction in LGR-5 expression in tumors treated with ACE. Although the results align with our expectations, the observed reduction was not statistically significant.
