# Supplementary material for: Areca catechu L. Extract Inhibits Colorectal Cancer Tumor Growth by Modulating Cell Apoptosis and Autophagy
Source: Curr Issues Mol Biol. 2025 Feb 17;47(2):128. doi: 10.3390/cimb47020128 (PMC11854706; doi:10.3390/cimb47020128)
Supplement: Supplementary file 1 [file cimb-47-00128-s001.zip › supplementary file/S6 Affidavit of Approval of Animal Use Protocol.pdf]

國立中央大學

實驗動物照護及使用委員會審查同意書

Affidavit of Approval of Animal Use Protocol

National Central University

同意書編號：NCU-110-011

計畫主持人(PI)：蘇立仁　　職　稱：副教授

單　　位：生醫系

飼養地點：生科系動物房　　應用地點：

計畫名稱：驗證傳統中藥 BP025W 對於體內大腸直腸癌腫瘤的毒殺效果

動物實驗申請表編號：

本計畫之「動物實驗申請表」業經實驗動物照護及使用委員 會審查通過。
本計畫預定飼養應用之動物如下：

動物種別	動物數量	計畫執行期間
CAnN.Cg-*Foxn1*^{nu}/CrlNarl	30 隻	110 年 5 月 1 日至 110 年 8 月 31 日

The animal use protocol listed below has been reviewed and approved by the Institutional Animal Care and Use Committee（IACUC）

Protocol Title：

IACUC Approval No：

Period of Protocol：Valid From: / / To: / / （mm/dd/yyyy）

Principal Investigator（PI）：

國立中央大學
實驗動物照護及使用委員會
主任委員 蔡振瀛

實驗動物照護及使用委員會召集人　　日期 110. 5. 7

IACUC Chairman　　Date
