# Supplementary material for: Areca catechu L. Extract Inhibits Colorectal Cancer Tumor Growth by Modulating Cell Apoptosis and Autophagy
Source: Curr Issues Mol Biol. 2025 Feb 17;47(2):128. doi: 10.3390/cimb47020128 (PMC11854706; doi:10.3390/cimb47020128)

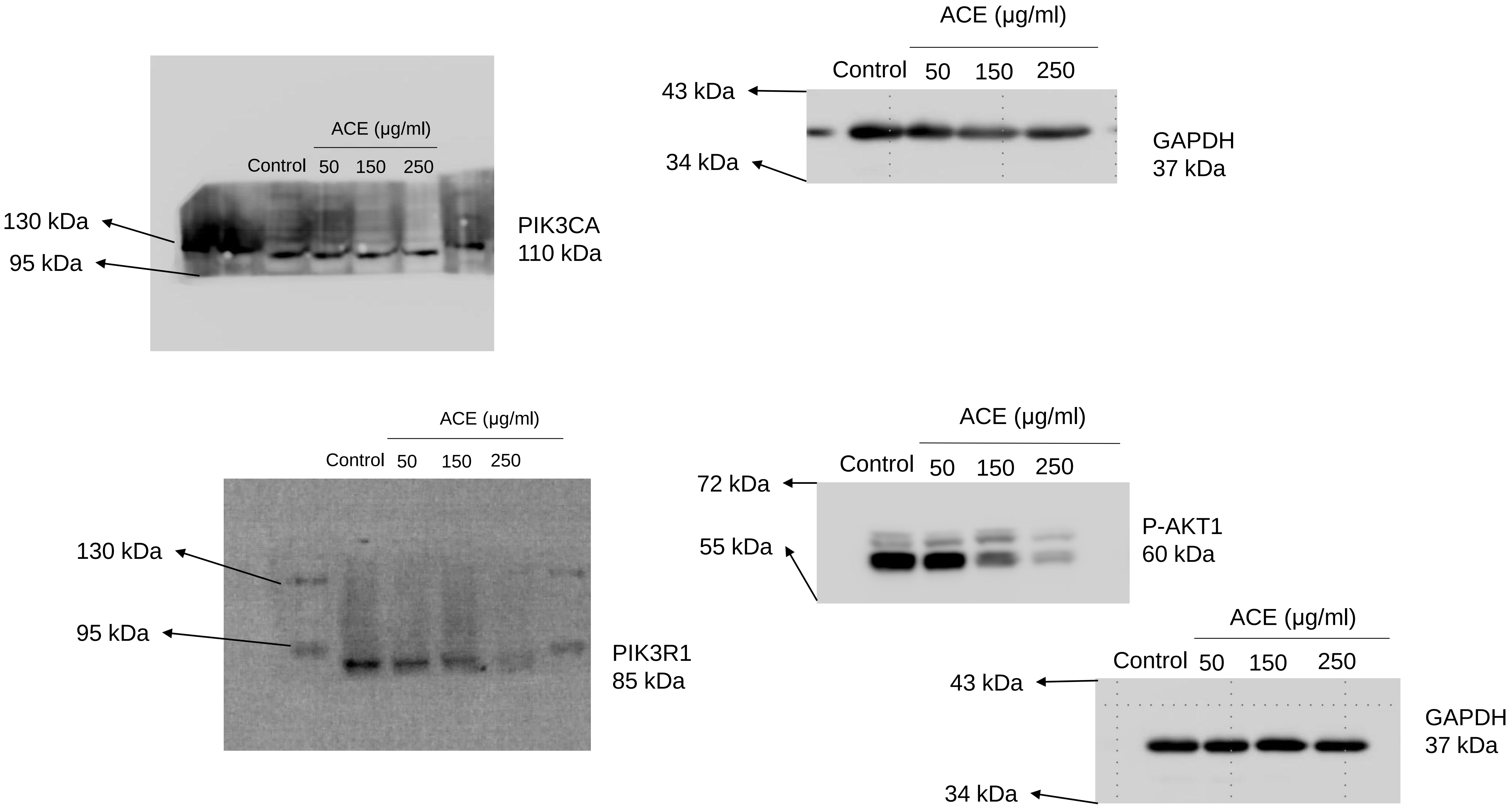

ACE (μg/ml)
Control 50 150 250
130 kDa
95 kDa
PIK3CA
110 kDa
ACE (μg/ml)
Control 50 150 250
43 kDa
34 kDa
GAPDH
37 kDa
ACE (μg/ml)
Control 50 150 250
130 kDa
95 kDa
PIK3R1
85 kDa
ACE (μg/ml)
Control 50 150 250
72 kDa
55 kDa
P-AKT1
60 kDa
ACE (μg/ml)
Control 50 150 250
43 kDa
34 kDa
GAPDH
37 kDa

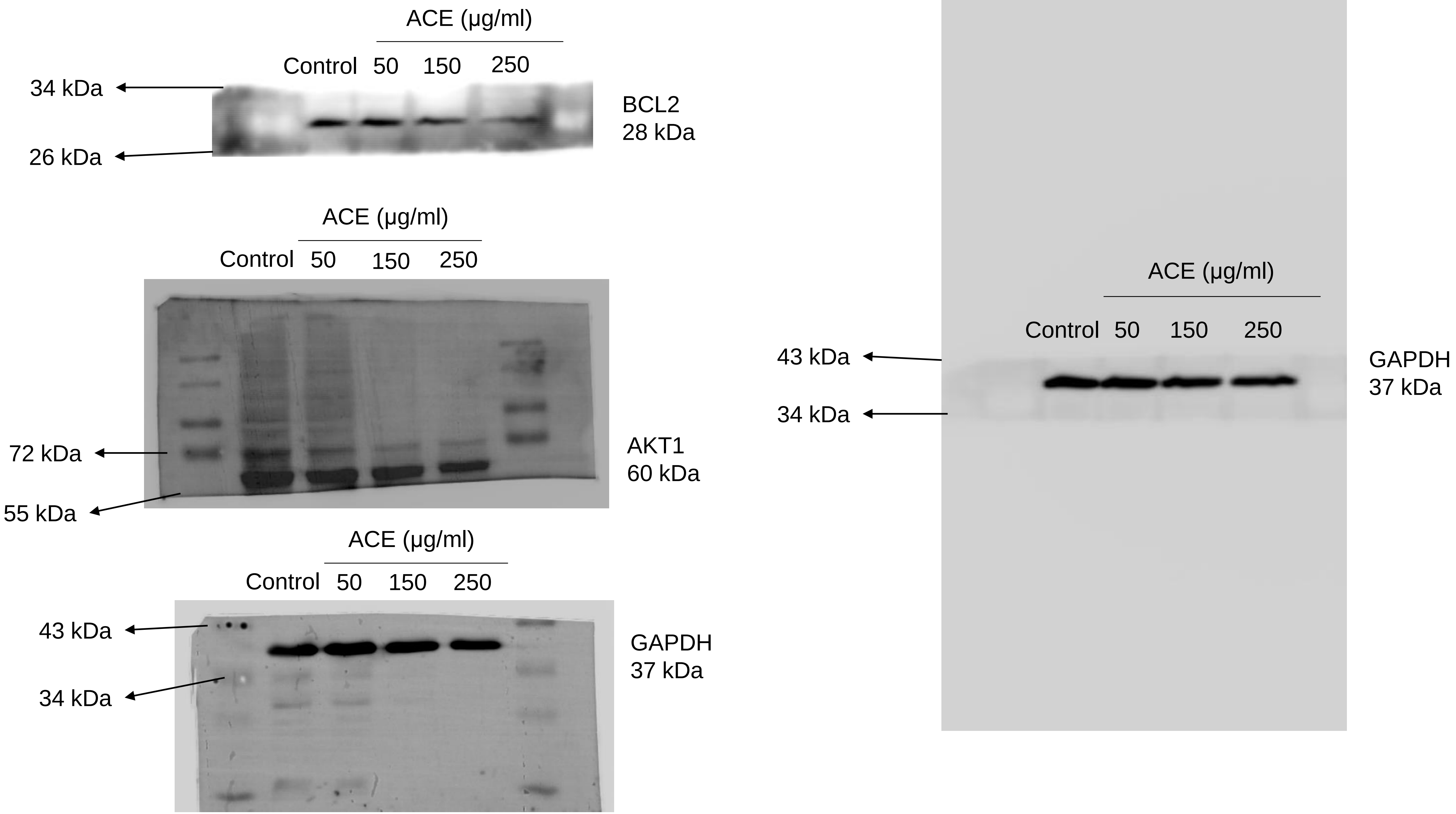
ACE (µg/ml)
Control 50 150 250
34 kDa
26 kDa
BCL2
28 kDa
ACE (µg/ml)
Control 50 150 250
72 kDa
55 kDa
AKT1
60 kDa
ACE (µg/ml)
Control 50 150 250
43 kDa
34 kDa
GAPDH
37 kDa
ACE (µg/ml)
Control 50 150 250
43 kDa
34 kDa
GAPDH
37 kDa

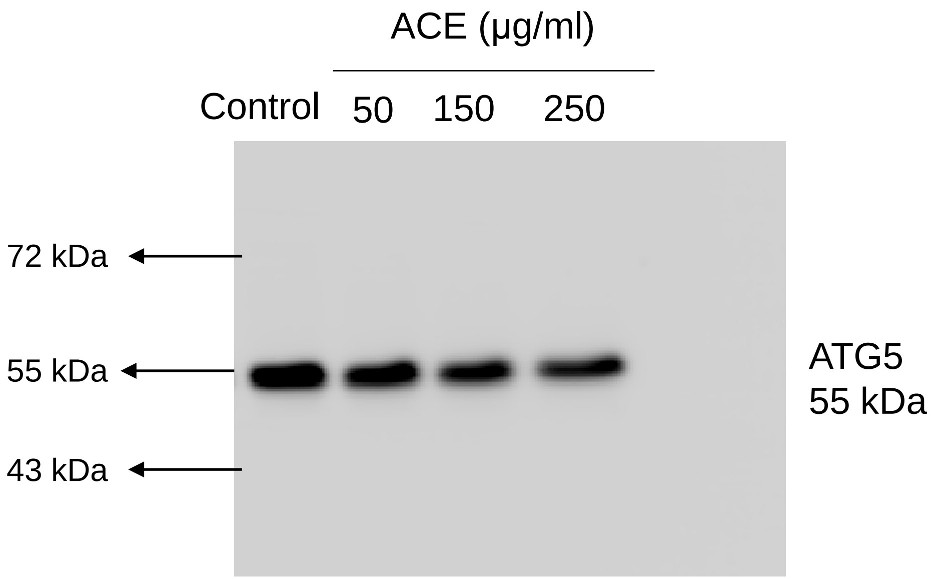
ACE (µg/ml)
Control
50
150
250
72 kDa
55 kDa
43 kDa
ATG5
55 kDa

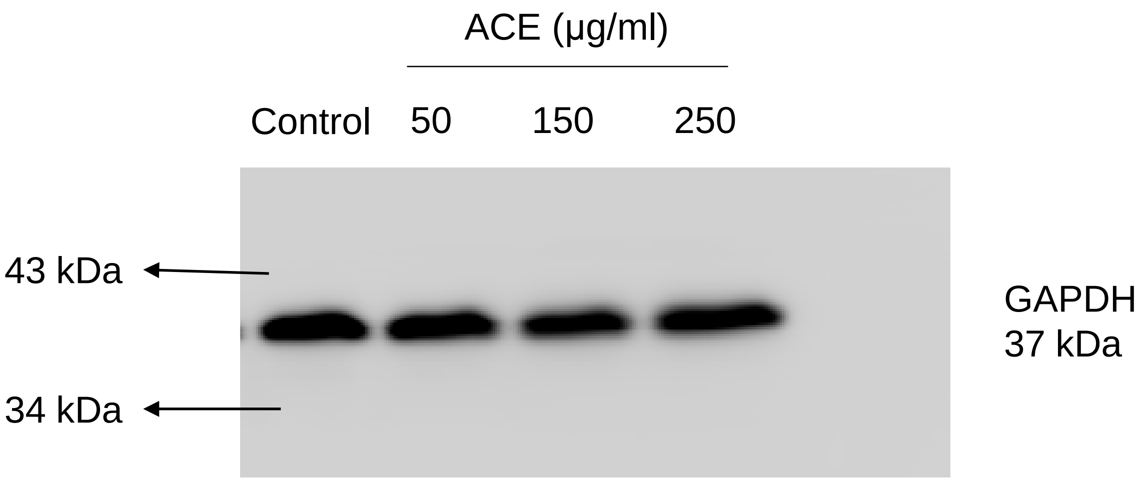
ACE (µg/ml)
Control
50
150
250
43 kDa
34 kDa
GAPDH
37 kDa

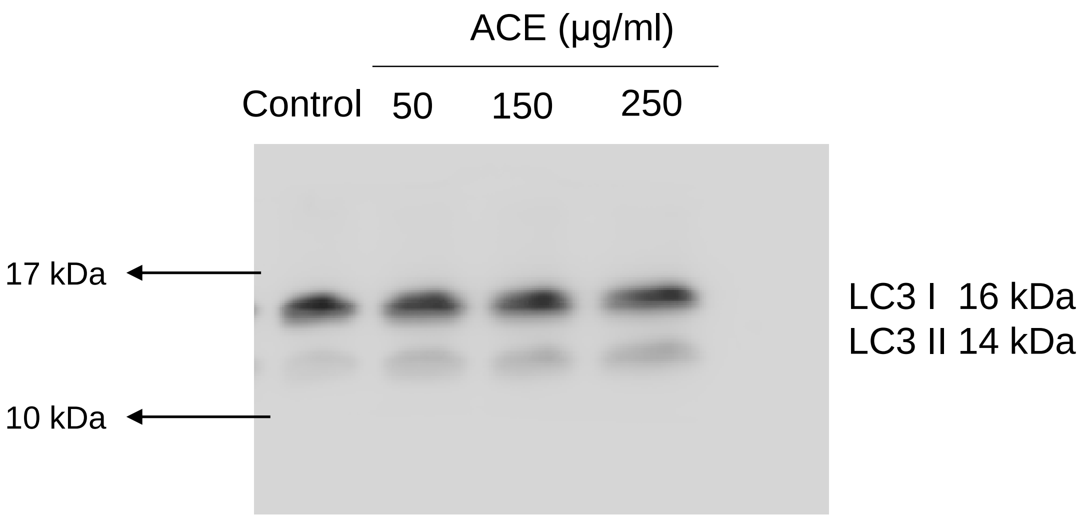
ACE (µg/ml)
Control
50
150
250
17 kDa
10 kDa
LC3 I 16 kDa
LC3 II 14 kDa

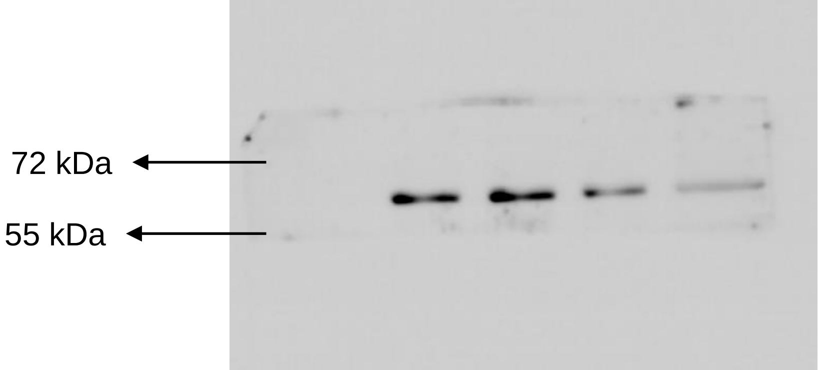

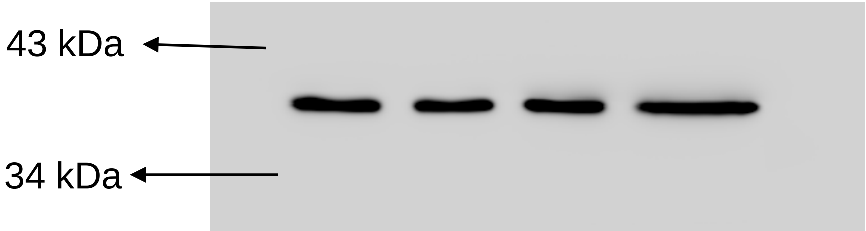

Used the primary antibody for GAPDH to detect the protein expression levels first. After detection, striped the GAPDH primary antibody using a stripping buffer, and then use the primary antibody for ATG3 to detect the protein expression levels.

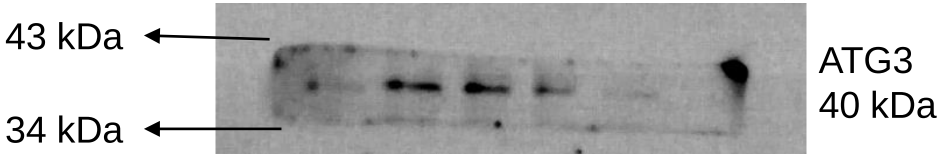

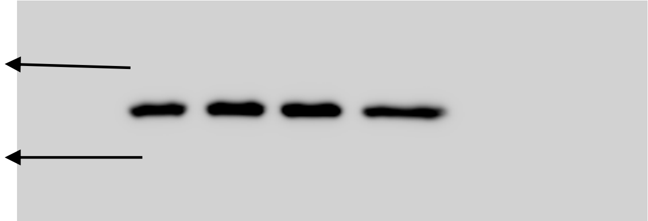

Supplement: Supplementary file 1 [file cimb-47-00128-s001.zip › supplementary file/S7 Western blot row image.pdf]
